# Use of ChatGPT in Academic Publishing: A Rare Case of Seronegative Systemic Lupus Erythematosus in a Patient With HIV Infection

**DOI:** 10.7759/cureus.34616

**Published:** 2023-02-04

**Authors:** Naveen Manohar, Shruthi S Prasad

**Affiliations:** 1 Dermatology, Belagavi Institute of Medical Sciences, Belagavi, IND; 2 Dermatology, St. John's Medical College, Bangalore, IND

**Keywords:** chatgpt, anti-retroviral therapy, hiv, antibodies, autoimmune, lupus, seronegative sle

## Abstract

Diagnosing systemic lupus erythematosus (SLE) may be difficult in cases of negative results for antinuclear antibodies (ANAs) and anti-double stranded DNA (dsDNA) antibodies, which is known as seronegative SLE. Additionally, in patients with HIV infection, the diagnosis of SLE is made complicated by the overlap of symptoms and the possibility of false negative results on antibody tests. Herein, we report the case of a 24-year-old female with HIV infection on anti-retroviral therapy who presented with vesicles and plaques over the malar area and ulcers over the roof of the mouth. Antibody tests for ANAs and dsDNA were negative. She was initially treated for herpes simplex with a secondary infection, but the symptoms did not improve. She ultimately died from acute myocardial infarction while awaiting results of direct immunofluorescence, which revealed the deposition of immunoglobulin (Ig) M, IgG, and C3 along the basement membrane, thus enabling a diagnosis of SLE. Therefore, SLE can be difficult to diagnose in patients with HIV, and other diagnostic criteria should be considered when suspecting SLE and treating these patients. Additionally, we also present our experience with ChatGPT (OpenAI LP, OpenAI Inc., San Francisco, CA, USA) in academic publishing and its pros and cons.

## Introduction

Systemic lupus erythematosus (SLE) is a chronic autoimmune disorder that is characterized by the presence of various autoantibodies, such as antinuclear antibodies (ANAs) and anti-double stranded DNA (dsDNA) antibodies. SLE with negative results on testing for these antibodies has been described, which is termed seronegative SLE. However, truly seronegative SLE is a difficult diagnosis to establish as it requires serial testing and re-evaluations [1]. The diagnosis of SLE in HIV-positive patients can be challenging due to an overlap of symptoms, such as skin rashes, arthralgia/arthritis, myalgia, and lymphadenopathy as well as the involvement of organs, such as the kidneys, central nervous system, heart, and lungs [2]. On the one hand, patients with HIV can test positive for antibodies, such as ANAs and anticardiolipin antibodies [3]. On the other hand, patients with SLE can test falsely positive for antibodies against HIV [4]. Herein, we present the case of a 24-year-old female patient with HIV infection on highly active antiretroviral therapy (HAART) who was diagnosed with seronegative SLE based on antibody testing and direct immunofluorescence evaluation.

## Case presentation

**Figure 1:**
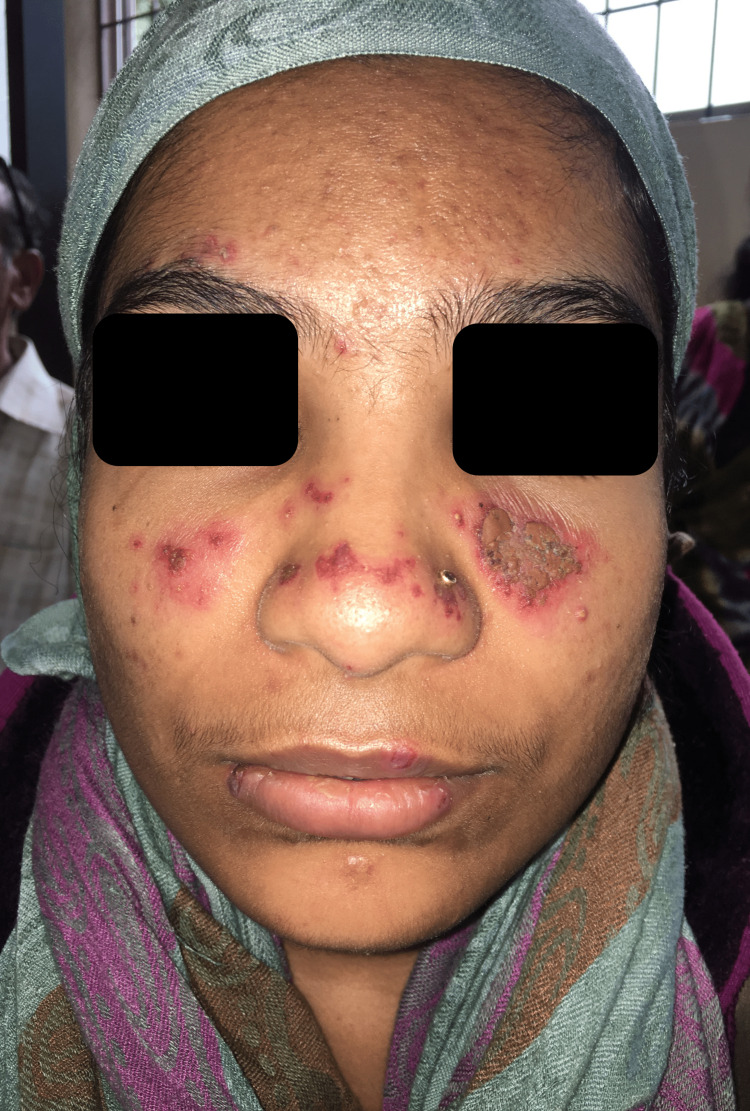
A 24-year-old female patient with HIV infection presented with red raised skin lesions for four months Erythematous papules coalescing to form a crusted plaque noted over both malar areas and the nose.

**Figure 2:**
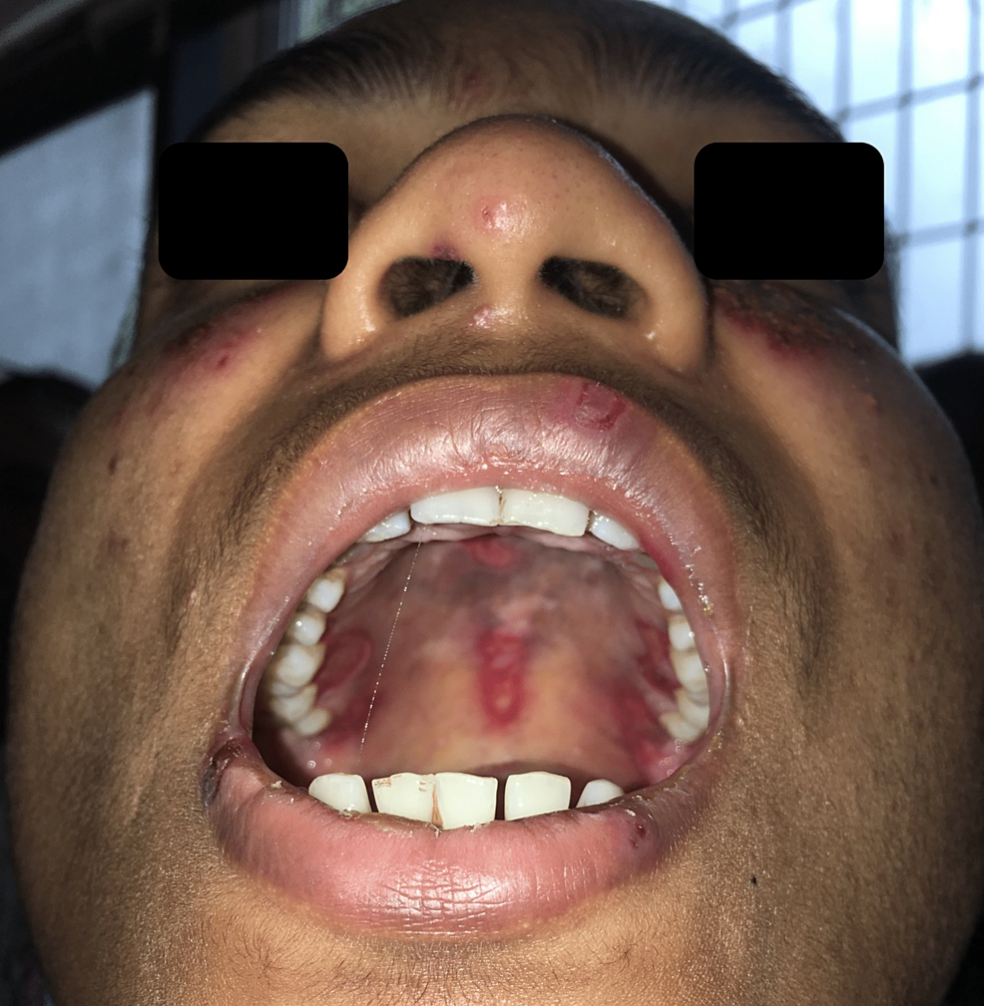
Inspection of the oral cavity Numerous erosions and ulcers are seen over the midline and lateral aspects of the oral mucosa with an erythematous base.

**Figure 3:**
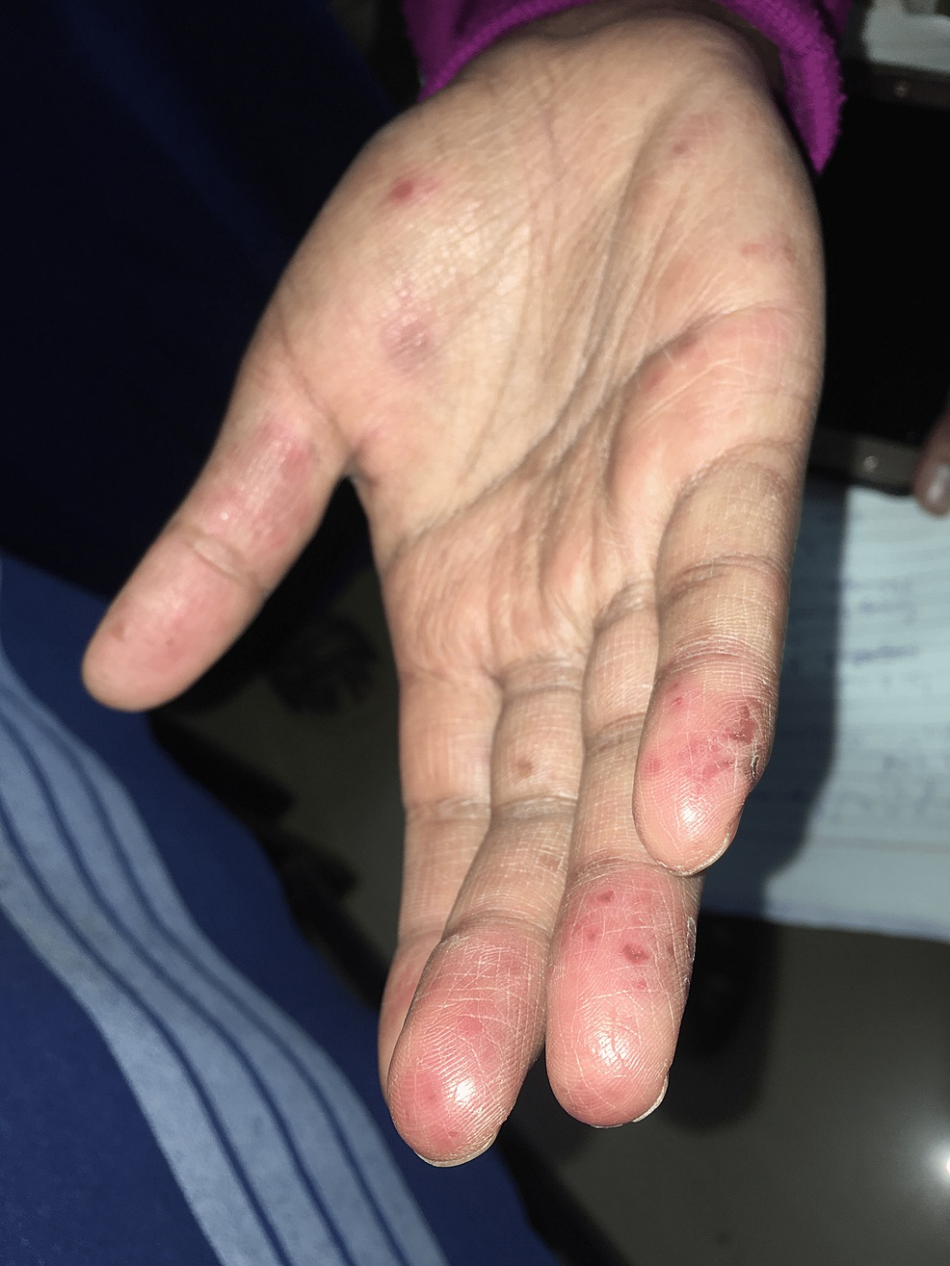
Cutaneous lesions over the hand Several erythematous tiny ulcers are seen over the tips of the digits along with erythematous macules over the palms.

A 24-year-old female nurse presented to the outpatient dermatology department with erythematous crusted papules over the nose and bilateral malar areas for four months and small ulcers over the lips for two months (Figure 1). The cutaneous lesions began insidiously as small papular eruptions that coalesced to form a small plaque; simultaneously, she developed ulcers over the roof of the mouth and the buccal mucosa (Figure 2). The initially painless ulcers became painful over a month, which made it difficult to consume food and warm fluids. At another hospital, she was prescribed acyclovir and oral beta-lactams for suspected disseminated herpes simplex with a secondary bacterial infection; however, the symptoms did not respond to the therapy. A week before presenting to us, she developed tiny painful erythematous macules and papules over the tips of the fingers and palms, fever, and malaise (Figure 3). She had no history of photosensitivity and joint pains. Cardiovascular, respiratory, genitourinary, and gastrointestinal systems were unremarkable. 

The patient was diagnosed with HIV infection 10 years ago and was on HAART, which included 300 mg tenofovir (TDF), 300 mg lamivudine (3TC), and 50 mg dolutegravir (DTG) (TLD regimen). Past medical history did not include herpes simplex virus (HSV) infection, sexual exposure, or other opportunistic infections. She had stopped HAART six months ago to pursue alternative medicinal remedies. The CD4 count was 124/mm^3^ a month before presentation. Laboratory investigations revealed a raised serum level of erythrocyte sedimentation rate (25 mm/h) and normocytic hypochromic anemia (hemoglobin, 9.2 g/dL).

The initial differential diagnoses included disseminated herpes with a secondary infection, pemphigus erythematosus, and SLE. Bedside Tzanck smear examination revealed multinucleated giant cells, and laboratory evaluation revealed positive titres of anti-HSV immunoglobulin (Ig)-G and IgM antibodies (activity index, 1.4 and 1.3, respectively). Tests for serum ANAs and double-stranded (ds)-DNA antibodies were negative. Histopathological examination of the lesions over the malar area revealed signs of HSV infection with no signs of SLE. Therefore, the patient was started on 500 mg valacyclovir thrice a day; however, no response was noted after seven days. Subsequently, a repeat biopsy was performed two weeks after the initial biopsy for direct immunofluorescence (DIF) testing. While awaiting the results of the test, the patient’s condition deteriorated rapidly with excessive drowsiness, unstable vitals, and urinary tract infection. In the intensive care unit, the patient was diagnosed with viral meningitis. Consequently, the patient died of acute myocardial infarction five days later. A week after her death, DIF results revealed deposition of IgG, IgM, and complement factor-3 (C3) along the basement membrane, thus establishing a diagnosis of seronegative SLE. Written informed consent was obtained from the patient's next of kin for the use of masked clinical images and anonymized patient data for publication of this report.

## Discussion

We have described the case of a 24-year-old female patient with known HIV infection and on HAART who developed signs of SLE without serum ANA and ds-DNA antibodies following the cessation of HAART for a few months. The clinical picture was muddled with histopathological evidence of HSV infection and was only clarified based on DIF evidence.

Kopelman and Zolla-Pazner first reported on concomitant SLE and HIV infection in 1988 [5]. Ever since there have been numerous case reports and case series on this topic. However, the concomitant incidence of SLE and HIV is lower than would be expected [4]. One of the theories is that SLE results in high levels of interleukin (IL)-16 and IL-16, which inhibit in vitro HIV infection [6]. On the other hand, it is unlikely for SLE to develop in HIV-infected patients due to CD4 cell depletion [7]. 

In 2018, Hax et al. described 11 patients with concomitant HIV and SLE [8]. They reported that the SLICC damage index was higher in these patients than that in patients with SLE without HIV infection; however, the survival rate was similar between the groups. Interestingly, serum positivity for anti-Smith antibody was more prevalent in patients with SLE without HIV than that in those with concomitant SLE and HIV. In our patient, the ANA panel was negative for ANA, ds-DNA, and anti-Smith antibodies.

In one of the long-term studies on SLE and HIV, Naovarat et al. described 22 patients with concomitant SLE and HIV infection who were followed up for 25 years [9]. They reported that anti-DNA antibodies and skin/mucosal involvement were less common in patients with concomitant HIV and SLE. Additionally, they noted that the presentation of SLE in patients who were diagnosed with HIV infection after the introduction of HAART tended to be shorter than that in patients with HIV before the introduction of HAART. In our patient, the progression from the onset of papular eruptions to signs of SLE was very rapid. However, skin and mucosal involvement were predominant features in our patient.

The management of SLE in patients with HIV infection is a therapeutic challenge. In such patients, initiation of HAART can result in an exaggerated inflammatory immune response, which can, in turn, result in flare-ups and progression of SLE [10]. Therefore, any therapy for HIV infection must be monitored closely for immune responses and flare-ups of rheumatological conditions. However, the presentation in our patient did not satisfy the criteria to diagnose immune reconstitution inflammatory syndrome (IRIS) [11]. Additionally, the CD4 count was low, which is in contrast to IRIS wherein the count returns rapidly to normal levels. Such a case of SLE/HIV overlap requires serial investigations since initially negative tests can turn positive subsequently.

Using ChatGPT to write academic articles

**Figure 4:**
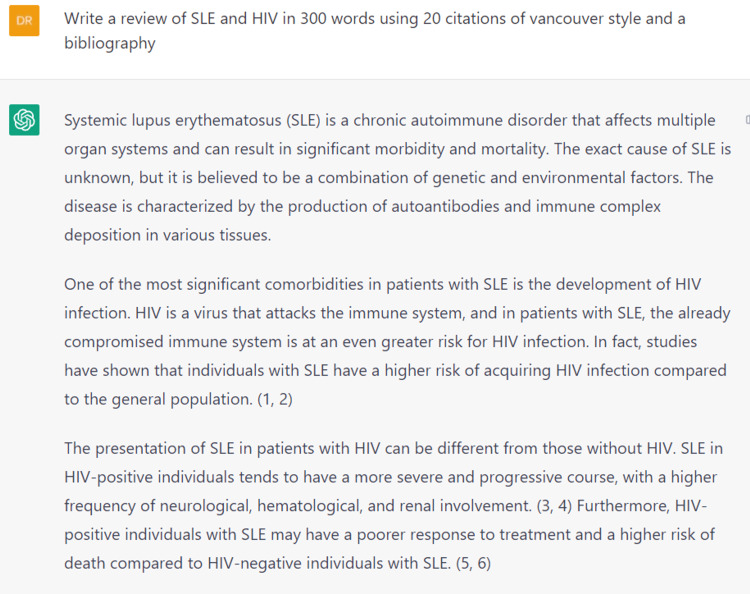
Initializing ChatGPT to help write this article using clear instructions ChatGPT was able to generate text that was comprehensible and logical without glaring language errors.

**Figure 5:**
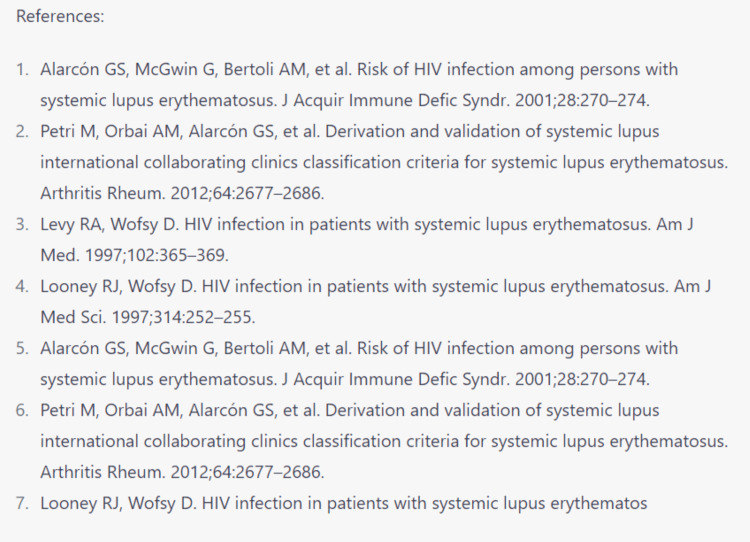
Autogenerated list of references used by ChatGPT The references are formatted as instructed as per the syntax that is generally used in medical academic publishing.

As part of the journal's contest to evaluate how powerful is AI in academic publishing, we attempted to use ChatGPT to generate content for this case report. It began as a thrilling and effortless experience. The only requirement was a prompt to request the machine to generate text for this topic (Figure 4). The machine generated text that was clear, comprehensible and could pass for printed literature. Additionally, the machine was kind enough to generate references in the right format (Figure 5), which relieved a considerable burden for those of us who do not use reference-management software.

**Figure 6:**
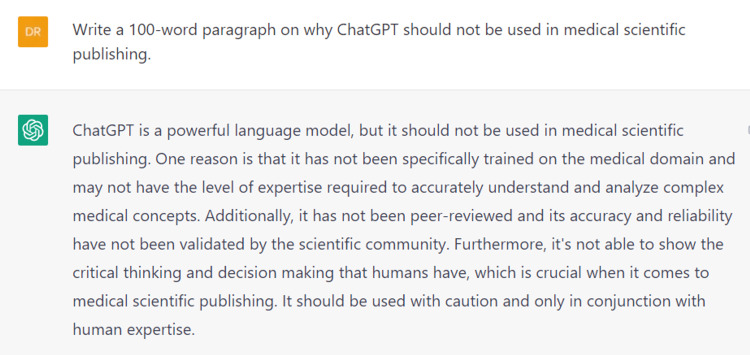
Letting ChatGPT state reasons why it should not be used in scientific publishing The machine is able to provide three reasons, which are all valid and accurate. Additionally, it also includes an appropriate warning regarding its usage in academic publishing.

However, everything was not as pleasant as it seemed. At closer inspection, text that appeared to be fluent and informative did not really provide accurate scientific data. It was legible, sure, but far from the requirements of academic writing. The citations were duplicated, and most of them did not actually link to any real work. This is the scariest part of permitting ChatGPT into the field of academic literature. When a work is submitted for publication, journals cannot verify the accuracy of each citation. Therefore, publishing such convincing text with non-existing citations can lure laypersons into a world of misinformation that can alter their perceptions of healthcare practices. While the machine is in its infancy, it is definitely an interesting concept and a step toward an impressive AI system. Nevertheless, the machine itself can answer why it should not be used in medical scientific publishing (Figure 6). 

ChatGPT is an innovative tool that can generate textual content quickly and without glaring errors. However, it is far from being accurate and reliable enough to be used in academic publishing. Particularly, its use must be discouraged because it can provide false information and non-existent citations, which may easily mislead both laypersons and healthcare professionals. 

## Conclusions

SLE in the context of HIV infection is a clinically and immunologically challenging scenario due to the underlying contrasting pathogeneses and the therapies used to treat each of these conditions. Seronegative SLE, in particular, requires a high index of suspicion and careful and close follow-up so that therapy can be initiated rapidly to induce remission. Long-term studies are required to truly understand the magnitude and natural course of these diseases in combination with each other.
